# The Influence of Achievement Motivation on Nurses’ Health‐Related Procrastination: The Mediating Role of Social Support

**DOI:** 10.1155/jonm/3802852

**Published:** 2026-04-30

**Authors:** Haili Zhang, Yi Sheng, Meiqin Hu, Liangyi Cao, Ling Xu, Lan Chen

**Affiliations:** ^1^ School of Nursing, Shanghai Jiao Tong University, Shanghai, China, sjtu.edu.cn; ^2^ Nursing Department, Shanghai General Hospital, Shanghai, China

**Keywords:** achievement motivation, health-related procrastination, mediating effect, nurse, procrastination, social support

## Abstract

**Aims:**

The primary aim of this study is to examine the mechanisms linking achievement motivation (including motive for success (MS) and motive to avoid failure (MF)) and social support to nurses’ health‐related procrastination (NHRP) and to determine whether social support plays a mediating role in this relationship.

**Background:**

The health of nurses has long been a subject of widespread concern. Compared with general procrastination, NHRP is more directly linked to their health issues, which not only harms their own well‐being but may also affect nursing quality and patient safety. Exploring its intrinsic mechanisms with achievement motivation and social support from the perspective of positive psychology is essential for developing effective strategies to reduce NHRP and serves as a cornerstone for ensuring nursing quality and patient safety.

**Methods:**

From February to July 2025, 346 samples of nurses from first‐class comprehensive hospital in Shanghai were selected as participants. The mediating effect of social support on achievement motivation and the NHRP was investigated via the General Data Questionnaire, Nurses’ Health‐related Procrastination Scale, Social Support Rating Scale, and Achievement Motivation Scale. The mediation effects were tested using the PROCESS macro (Version 4.0), which utilizes linear regression analysis and the bootstrap method.

**Results:**

A total of 320 valid questionnaires were collected, yielding an effective response rate of 92.47%. The results indicated that social support was positively correlated with MS (*r* = 0.339, *p* < 0.01) and negatively correlated with both MF (*r* = −0.283, *p* < 0.01) and NHRP (*r* = −0.482, *p* < 0.01). Furthermore, NHRP was negatively correlated with MS (*r* = −0.433, *p* < 0.01) and positively correlated with MF (*r* = 0.397, *p* < 0.01). Notably, social support was found to partially mediate the relationship between achievement motivation and NHRP. The specific mediating effects of social support were significant, with indirect effect values of −0.468 between MS and NHRP and 0.422 between MF and NHRP, accounting for 31.0% and 29.8% of the total effects, respectively.

**Conclusion:**

Achievement motivation can directly influence NHRP and can also indirectly affect it through the mediating role of social support. From the perspective of positive psychology, stimulating nurses’ achievement motivation regarding their own health responsibility and fostering a supportive organizational atmosphere can effectively reduce their health‐related procrastination, thereby laying a foundation for enhancing overall nursing service quality.

**Implications for Nursing Management:**

Nursing managers should pay attention to nurses’ physical and mental health and actively identify and guide the orientation of their achievement motivation: on the one hand, cultivate their positive motivation for “striving for success,” and on the other hand, alleviate the anxiety stemming from “fear of failure,” thereby fostering the formation of health behaviors through intrinsic mechanisms. Furthermore, it is essential to consciously build a team atmosphere with high social support and to reduce NHRP by establishing effective peer support systems and communication channels.

## 1. Introduction

Nurses, as a core force within the healthcare system, play a vital role in health education and promotion. Their own health serves not only as a prerequisite for effective health education efforts but also as a critical factor in enhancing the quality of nursing care and ensuring patient safety [1]. However, the nature of nursing work—characterized by high workload, frequent emotional labor, significant occupational exposure risks, and shift work—subjects nurses to various health threats [2–4]. Data indicate that the prevalence of subhealth among Chinese nurses is as high as 74.21% [5], with a chronic disease prevalence of 33.5% [6]. The health crises faced by nurses themselves exacerbate the existing shortage of nursing staff, creating a vicious cycle. Notably, a phenomenon of “separation of knowledge, belief, and practice” is common among nurses, meaning that despite possessing health knowledge, they often delay or neglect their own health management in practice [7]. Recent studies suggest that nurses may employ ineffective coping mechanisms in response to work‐related stressors, with behaviors such as irrational procrastination, work procrastination, and bedtime procrastination garnering increasing academic attention [8, 9]. Compared with general procrastination, health‐related procrastination has a stronger and more direct association with health outcomes. It refers to the voluntary and unnecessary delay in enacting health behaviors or tasks, despite having an initial intention to start or change them [10]. This behavior is a key catalyst in the progression from subhealth to disease, and reducing health‐related procrastination can significantly improve the health status. Nevertheless, current research both domestically and internationally predominantly focuses on cross‐sectional surveys of nurses’ general procrastination or their health‐promoting lifestyles [11, 12]. There remains a paucity of targeted research that integrates these aspects to specifically focus on health‐related procrastination among nurses.

To address this research gap, this study employs Bandura’s “Triadic Reciprocal Determinism” as the theoretical framework, adopting a positive psychology perspective. This theory posits that the individual, the environment, and behavior constitute a dynamic, interacting determinative system [13]. In this study, achievement motivation is regarded as a key personal cognitive factor, social support represents a crucial environmental factor, and nurses’ health‐related procrastination (NHRP) is the specific behavioral outcome under investigation.

First, achievement motivation is an intrinsic driver for individuals to pursue goals and take action, and it is an important construct in positive psychology [14]. Achievement motivation theory indicates that individual behavior is primarily driven by two dimensions: the “motive for success (MS)” and the “motive to avoid failure (MF)” [15]. Research shows that nurses’ procrastination is negatively correlated with their MS and positively correlated with their MF [16]. This may be because pursuing goals perceived as important and valuable can stimulate individual agency, thereby reducing procrastination, whereas individuals with high levels of failure‐avoidance motivation tend to delay starting or completing tasks due to anxiety or self‐doubt [17]. Other studies have shown that stronger motives for success among clinical nurses are associated with better mental health outcomes [18]. Although direct empirical research on the relationship between achievement motivation and health‐related procrastination remains scarce, based on the aforementioned theory and empirical evidence, this study posits that achievement motivation may have a direct association with NHRP.

Second, social support, as a core positive resource in the environmental context, refers to the emotional support, informational support, and tangible assistance available to an individual [19]. Studies indicate that social support serves as a protective factor that can buffer the negative impact of stressors. A higher level of social support helps individuals build psychological capital, thereby reducing the occurrence of behaviors such as procrastination [20]. It also enhances individuals’ health management capabilities and promotes health‐promoting lifestyles [21]. Therefore, this study proposes that social support may be directly associated with NHRP.

Finally, an individual’s intrinsic traits can also elicit different environmental responses. Most studies suggest that a high MS often facilitates the perception and active seeking of environmental support, while individuals characterized by a high MF may reduce proactive help‐seeking behaviors due to fear of negative evaluation or self‐doubt [22, 23]. Additionally, individuals with high achievement motivation often possess greater self‐confidence and self‐control, believing that they can overcome challenges through their own efforts and external support and are, therefore, more likely to proactively seek assistance [24].

**FIGURE 1:**
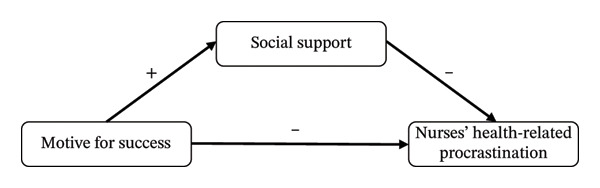
Hypothesized research framework 1.

**FIGURE 2:**
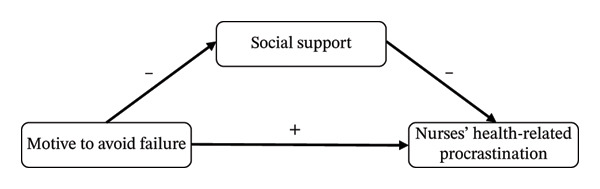
Hypothesized research framework 2. Note: ^∗∗∗^ *p* < 0.001.

In summary, based on the theory of triadic reciprocal determinism and an analysis of the interrelationships among the variables discussed above, this study constructs a parallel mediation model with social support as the mediator (Figures 1 and 2). This model aims to systematically elucidate the underlying mechanisms linking achievement motivation, social support, and NHRP, with a specific focus on the mediating role of social support. From a positive psychology perspective, this study seeks to provide a theoretical foundation for strengthening nurses’ health management and improving the quality of nursing care. Accordingly, the following research hypotheses are proposed: H1: The MS is negatively correlated with NHRP, whereas the MF is positively correlated with NHRP. H2: The MS is positively correlated with social support, whereas the motive to avoid failure is negatively correlated with social support. H3: Social support is negatively correlated with NHRP. H4: Social support mediates the relationship between the MS and NHRP. Simultaneously, social support also mediates the relationship between the MF and NHRP.

## 2. Methods

### 2.1. Study Design

The study used a cross‐sectional descriptive design that can measure multiple variables at the same time, facilitating the evaluation of the relationships among different variables. This study adopted the STROBE guidelines [25] to ensure the sufficiency and completeness of the research report.

### 2.2. Participants

Nurses from a Grade Three general hospital in Shanghai were selected as the study participants from February to July 2025 via the convenience sampling method. The inclusion criteria for participants were as follows: (1) provision of informed consent; (2) possession of a valid nurse practicing certificate; (3) engagement in clinical nursing work for at least 12 months; and (4) being on active duty during the data collection period. Exclusion criteria included trainee and student nurses. Sample size was determined using *G* × Power 3.1 software. Based on the tested Hayes PROCESS Model 4 for simple mediation, with a conservative small effect size (*f* ^2^ = 0.10), *a* = 0.05, and *power* = 0.80, the minimum required sample size was calculated to be 107. Accounting for a 20% rate of invalid responses, this study ultimately collected 320 valid samples. This study was reviewed by the Medical Ethics Committee of Shanghai General Hospital (no. 2025‐038). All the subjects were aware of this study and voluntarily participated in the investigation.

### 2.3. Instruments

#### 2.3.1. General Information Questionnaire

Based on relevant literature [26, 27], and the actual conditions of the institution as well as expert opinions, the questionnaire was self‐designed by the researchers and covered 10 items including gender, age, type of clinical unit, education level, marital status, parental status, professional title, years of working, average monthly income, and certified specialist nurse.

#### 2.3.2. The Nurses’ Health‐Related Procrastination Scale (NHRPS)

This scale was developed by Basirimoghadam et al. [28] and later translated and adapted into Chinese by Zhang et al. to assess the level of health‐related procrastination among nurses [29]. The Chinese version of the NHRPS consists of 25 items across four dimensions: procrastination in maintaining physical health (8 items), procrastination in promoting physical health (4 items), procrastination in social and mental health (9 items), and procrastination in spiritual health (4 items) (see Supporting Table 1). Responses are recorded on a 5‐point Likert scale ranging from “*Never*” (1 point) to “*Always*” (5 points). The total score ranges from 25 to 125, with higher scores indicating a greater degree of health‐related procrastination. The Cronbach’s *α* coefficient for this scale was 0.930 in the original validation, and it was 0.939 in the present study.

#### 2.3.3. The Achievement Motivation Scale (AMS)

This scale was developed by Norwegian psychologists Gjesme [30]. This study employed the Chinese version revised by Ye and Hagtvet [31]. The scale consists of 30 items and comprises two subscales: MS and MF, each containing 15 items. Responses are rated on a 4‐point Likert scale ranging from “1 (*completely inconsistent*)” to “4 (*strongly consistent*).” The two subscales are scored separately, with a total possible score of 60 for each. Higher scores indicate a higher level of the respective motivation. Although this Chinese version was initially applied in student populations, the psychological construct it measures demonstrates cross‐population generalizability and has since been adopted and used in several nursing studies both in China and internationally [18, 22, 32]. In this study, the scale showed good reliability, with Cronbach’s *α* coefficients of 0.939 and 0.936 for the two subscales, respectively.

#### 2.3.4. The Social Support Rating Scale (SSRS)

This scale was developed by Professor Xiao [33]. The scale comprises 10 items across three dimensions: objective support (3 items), subjective support (4 items), and utilization of social support (3 items). Total scores range from 12 to 66 and are categorized into three levels: low (< 33), moderate (33–45), and high (> 45), with higher scores indicating stronger social support. In recent years, the SSRS has been widely used in nursing research, demonstrating good reliability and validity [22, 34]. In this study, its Cronbach’s *α* coefficient was 0.766.

### 2.4. Data Collection

After obtaining consent from the nursing department and the head nurses, questionnaires were distributed via the Questionnaire Star platform and WeChat. The survey method along with the inclusion and exclusion criteria were explained in detail to the distributors, and the principles of anonymity and confidentiality were emphasized. Participants could only proceed after fully understanding the purpose, significance, and precautions of the study and were permitted to complete the survey only once. Incomplete questionnaires could not be submitted. A total of 346 questionnaires were collected. After excluding unqualified ones (e.g., those with obviously consistent answers or a response time of less than 200 s), 320 valid questionnaires were retained, resulting in an effective response rate of 92.47%.

### 2.5. Quality Control

The questionnaires were distributed by nursing graduate students who had received uniform training. All investigators were familiar with the study’s purpose and the inclusion/exclusion criteria. Questionnaires with an overly short response time (< 200 s) or a clear patterned response were considered invalid. After removing invalid responses, data were entered using a two‐person, double‐entry method. The entered data were then proofread and tested for logical consistency to ensure accuracy.

### 2.6. Data Analysis

Data were analyzed using SPSS 27.0. Categorical variables were expressed as frequencies and percentages. Normality of continuous variables was assessed using the Shapiro–Wilk test [35] (see Supporting Information 2, Table 1). Based on the normality test results, normally distributed variables (including scores of the SSRS and the NHRPS) were described as mean ± standard deviation and compared between groups using independent *t*‐tests or ANOVA. Nonnormally distributed variables (including scores of the MS and MF subscales) were presented as median (interquartile range) and analyzed using Spearman’s rank correlation. To examine the mediating role of social support between achievement motivation and NHRP, the PROCESS macro (v4.0) was employed, conducting linear regression analysis with the Bootstrap method (5000 resamples, *α* = 0.05).

**TABLE 1:** General characteristics of participants (*n* = 320).

Nurse characteristics	Category	*N* (%)
Gender	Male	7 (2.2)
	Female	313 (97.8)

Age	≤ 25	49 (15.3)
	26–30	68 (21.3)
	31–40	144 (45.0)
	≥ 41	59 (18.4)

Type of clinical unit	Internal Medicine	68 (21.3)
	Surgery Department	67 (20.9)
	Obstetrics and Gynecology	40 (12.5)
	Pediatrics Department	23 (7.2)
	Emergency and ICU	81 (25.3)
	Other	41 (12.8)

Education level	Diploma in Nursing	49 (15.3)
	Bachelor Degree	260 (81.3)
	Master Degrees or above	11 (3.4)

Marital status	Married	227 (70.9)
	Unmarried	93 (29.1)

Parental status	Has children	206 (64.4)
	No children	114 (35.6)

Professional title	Nurse	47 (14.7)
	Nurse practitioner	173 (54.1)
	Nurse in charge	97 (30.3)
	Associate professor of nursing or above	3 (0.9)

Years of working	≤ 5	81 (25.3)
	6–10	68 (21.3)
	11–15	58 (18.1)
	16–20	72 (22.5)
	≥ 21	41 (12.8)

Average monthly income	≤ 5000	15 (4.7)
	5001–7000	31 (9.7)
	7001–9000	116 (36.3)
	9001–10000	105 (32.8)
	≥ 10,001	53 (16.6)

Certified specialist nurse	Yes	114 (35.6)
	No	206 (64.4)

### 2.7. Ethics Statement

This study was approved by the Medical Ethics Committee of Shanghai General Hospital (no. 2025‐038). Informed consent was obtained from all participants prior to their inclusion in the study. Participants were informed that they could withdraw at any time without penalty. All procedures were conducted in accordance with the ethical principles outlined in the Declaration of Helsinki.

## 3. Results

### 3.1. General Characteristics and Univariate Analysis of NHRP

This study included 320 valid questionnaires. The majority of participants were female (97.8%) and married (70.9%). The most common age group was 31–40 years (45.0%), and the largest proportion of nurses (25.3%) worked in the emergency department and intensive care unit (ICU). Overall, 81.3% of the nurses had a bachelor’s degree, and 54.1% held the professional title of nurse (the most junior professional title). The demographic characteristics of the nurses are shown in Table 1.

### 3.2. Scores of NHRP, Social Support, and Achievement Motivation

The score for NHRP was 61.33 ± 17.15, with the dimension of procrastination in maintaining physical health receiving the lowest score. The overall social support score was 37.93 ± 7.46, and among its dimensions, the utilization of social support scored the lowest. Regarding achievement motivation, the scores for the MS and the MF were 39.00 (31.00, 46.00) and 40.00 (34.25, 48.00), respectively. The detailed scores for each variable are presented in Table 2.

**TABLE 2:** Scores of the NHRP, social support, and achievement motivation (*n* = 320).

Variable	Average score	Item average score
Nurses’ health‐related procrastination	61.33 ± 17.15	2.45 ± 0.69
Procrastination in maintaining physical health	16.94 ± 6.60	2.12 ± 0.83
Procrastination in physical health promotion	12.20 ± 3.64	3.05 ± 0.91
Procrastination in social and mental health	21.26 ± 7.47	2.36 ± 0.83
Procrastination in spiritual health	10.93 ± 3.13	2.73 ± 0.78
Social support	37.93 ± 7.46	3.79 ± 0.75
Subjective support	22.20 ± 5.11	5.55 ± 1.28
Objective support	8.20 ± 1.91	2.73 ± 0.64
Utilization of social support	7.53 ± 1.97	2.51 ± 0.66

*Achievement motivation*		
Motive for success	39.00 (31.00,46.00)	2.60 (2.07,3.07)
Motive to avoid failure	40.00 (34.25, 48.00)	2.67 (2.28,3.20)

### 3.3. Correlation Analysis of NHRP, Social Support, and Achievement Motivation

Correlations among the dimensions of NHRP (A1–A4), social support (B1–B3), and achievement motivation (C1‐C2) are presented in Table 3. Spearman correlation analysis indicated that NHRP was significantly negatively correlated with MS (*r* = −0.433, *p* < 0.01) and significantly positively correlated with MF (*r* = 0.397, *p* < 0.01). Social support was significantly positively correlated with MS (*r* = 0.339, *p* < 0.01), significantly negatively correlated with MF (*r* = −0.283, *p* < 0.01), and significantly negatively correlated with NHRP (*r* = −0.482, *p* < 0.01). These results provide support for Hypotheses 1, 2, and 3.

**TABLE 3:** Correlation analysis of the NHRP, social support, and achievement motivation.

	NHRP	A1	A2	A3	A4	SS	B1	B2	B3	MS	MF
NHRP	1										
A1	0.81^∗∗^	1									
A2	0.75^∗∗^	0.45^∗∗^	1								
A3	0.86^∗∗^	0.61^∗∗^	0.52^∗∗^	1							
A4	0.78^∗∗^	0.54^∗∗^	0.43^∗∗^	0.62^∗∗^	1						
SS	−0.48^∗∗^	−0.34^∗∗^	−0.37^∗∗^	−0.48^∗∗^	−0.36^∗∗^	1					
B1	−0.37^∗∗^	−0.29^∗∗^	−0.27^∗∗^	−0.35^∗∗^	−0.29^∗∗^	0.77^∗∗^	1				
B2	−0.43^∗∗^	−0.30^∗∗^	−0.29^∗∗^	−0.46^∗∗^	−0.32^∗∗^	0.80^∗∗^	0.42^∗∗^	1			
B3	−0.35^∗∗^	−0.21^∗∗^	−0.32^∗∗^	−0.33^∗∗^	−0.27^∗∗^	0.81^∗∗^	0.45^∗∗^	0.52^∗∗^	1		
MS	−0.43^∗∗^	−0.30^∗∗^	−0.33^∗∗^	−0.40^∗∗^	−0.33^∗∗^	0.34^∗∗^	0.16^∗∗^	0.34^∗∗^	0.34^∗∗^	1	
MF	0.40^∗∗^	0.27^∗∗^	0.33^∗∗^	0.36^∗∗^	0.33^∗∗^	−0.28^∗∗^	−0.17^∗∗^	−0.26^∗∗^	−0.28^∗∗^	−0.25^∗∗^	1

*Note:* NHRP, nurses’ health‐related procrastination; A1, procrastination in maintaining physical health; A2, procrastination in physical health promotion; A3, procrastination in social and mental health; A4, procrastination in spiritual health; SS, social support; B1, subjective support; B2, objective support; B3, utilization of social support; MS, motive for success; MF, motive to avoid failure.

^∗∗^ *p* < 0.01.

### 3.4. The Mediating Role of Social Support

To control for potential confounding factors, univariate analysis was first performed with demographic variables as independent variables and NHRP as the dependent variable. Variables that showed statistically significant differences were converted into dummy variables and then entered into a linear regression model. The results indicated that age, years of working, and education level were the main predictors of NHRP (entry criterion: *p* < 0.05 and removal criterion: *p* > 0.10), collectively explaining 11.0% of the total variance, as shown in Table 4. These variables were subsequently included as covariates in the mediation model for control. The analysis process is detailed in Supporting Information 3 and Tables 1 and 2.

**TABLE 4:** Multiple linear regression analysis of nurses’ health‐related procrastination and demographic variables (*n* = 320).

Independent variable	**B**	**SE**	**B**	**t**	*p*	95%**C** **I**
Constant	74.033	2.581		28.69	< 0.001	68.956–79.111

*Age (≤ 25 = control group)*						
26–30	−7.473	2.811	−0.179	−2.659	0.008	−13.003∼‐1.943
31–40	−6.861	2.189	−0.199	−3.135	0.002	−11.167∼‐2.555

*Years of working (≤ 5 = control group)*						
≥ 21	−7.683	2.271	−0.224	−3.384	< 0.001	−12.15∼‐3.216

*Education level (Diploma in Nursing = control group)*						
Bachelor Degree	−4.822	2.419	−0.11	−1.993	0.047	−9.583∼‐0.062

*Note:* *R* ^2^ = 0.110, adjusted *R* ^2^ = 0.099, *F* = 9.779.

#### 3.4.1. Mediating Effect of Social Support Between MS and NHRP

**FIGURE 3:**
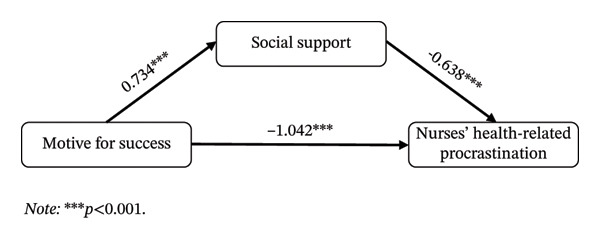
Mediating effect model 1. Note: ^∗∗∗^ *p* < 0.001.

As shown in Table 5, the total effect of MS on NHRP was −1.510, with a Bootstrap 95% CI of [‐1.916, −1.104] that did not include zero, indicating a significant total effect. The indirect effect through social support was −0.468, with a Bootstrap 95% CI of [‐0.700, −0.273]; the fact that this interval did not include zero suggests that social support played a significant partial mediating role between MS and NHRP. The indirect effect accounted for 31.0% of the total effect, while the direct effect accounted for 69.0%. The mediation model is illustrated in Figure 3.

**TABLE 5:** Mediating effect of social support between motive for success and nurses’ health‐related procrastination (unstandardized).

Model pathways	Estimate	SE	*p*	95% CI		Effect proportion
				Lower	Upper	
Indirect effect	−0.468	0.108	< 0.001	−0.700	−0.273	31.0%
Direct effect	−1.042	0.120	< 0.001	−1.435	−0.649	69.0%
Total effect	−1.510	0.206	< 0.001	−1.916	−1.104	

Abbreviations: CI = confidence interval, SE = standard error.

#### 3.4.2. Mediating Effect of Social Support Between MF and NHRP

**FIGURE 4:**
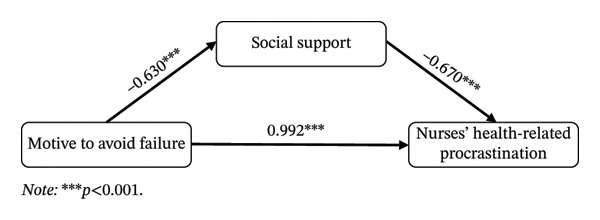
Mediating effect model 2. Note: ^∗∗∗^ *p* < 0.001.

As shown in Table 6, the total effect of MF on NHRP was 1.414, with a Bootstrap 95% CI of [0.988, 1.840] that did not include zero, indicating a significant total effect. The indirect effect through social support was 0.422, with a Bootstrap 95% CI of [0.226, 0.642]; since this interval did not include zero, social support played a significant partial mediating role between MF and NHRP. The indirect effect accounted for 29.8% of the total effect, while the direct effect accounted for 70.2%. The mediation model is illustrated in Figure 4. In summary, the results support Hypothesis 4.

**TABLE 6:** Mediating effect of social support between motive to avoid failure and nurses’ health‐related procrastination (unstandardized).

Model pathways	Estimate	SE	*p*	95% CI		Effect proportion
				Lower	Upper	
Indirect effect	0.422	0.107	< 0.001	0.226	0.642	29.8%
Direct effect	0.992	0.204	< 0.001	0.590	1.394	70.2%
Total effect	1.414	0.217	< 0.001	0.988	1.840	

Abbreviations: CI = confidence interval, SE = standard error.

## 4. Discussion

Grounded in this theoretical framework, the present study aimed to examine the relationships among nurses’ achievement motivation, social support, and health‐related procrastination and to investigate the mediating role of social support. The main findings underscore significant correlations among the variables and highlight the significant mediating effect of social support. These results are consistent with the hypotheses derived from triadic reciprocal determinism.

### 4.1. Status Analysis of Achievement Motivation, Social Support, and NHRP

The results of this study showed that nurses’ motivation to avoid failure (median = 40.0 and Q1–Q3: 34.3–48.0) was higher than their motivation to achieve success (median = 39.0 and Q1‐Q3: 31.0–46.0), which is consistent with the findings of Zhu et al. in their survey of medical and nursing students [36]. Due to the unique nature of the medical profession, which involves working with human lives, coupled with structural factors such as high clinical workload and insufficient staffing, nurses’ professional enthusiasm and intrinsic drive may be diminished, leading to a greater tendency to avoid failure [37]. Furthermore, the relatively low proportion of nurses with intermediate and senior professional titles in this study may also contribute to the lower level of achievement motivation. Professional title is an important marker of career development; nurses with lower titles typically have less autonomy and fewer opportunities to participate in significant decision‐making, which may constrain the development of their motivation to achieve success. In contrast, nurses with higher titles often possess greater work competence and richer clinical experience and correspondingly score higher in achievement motivation [22, 38]. This suggests that nursing managers should stimulate nurses’ achievement motivation by setting clear health goals and providing feedback and recognition, thereby promoting nurses’ proactive and timely adoption of health behaviors and reducing procrastination.

The results of this study showed that nurses’ social support was at a moderate level (37.87 ± 7.75), which is consistent with the findings of Zheng et al. regarding nurses in general wards [39], but lower than that reported by Shi et al. [40]. This discrepancy may be related to the relatively high proportion of junior nurses in the sample. Previous research has shown that junior nurses are still in the stage of professional role transition and adaptation, with their social networks not yet firmly established and their ability to seek and utilize support being relatively limited. This may lead to a lower overall perceived level of social support [41, 42]. Therefore, it is recommended that hospital managers and senior nurses actively foster a supportive team atmosphere and assist junior nurses in expanding and effectively utilizing their social support networks, thereby enhancing their willingness to implement health behaviors and reducing related procrastination.

The results of this study showed that NHRP was at a moderate level, indicating that this phenomenon is relatively common among the nursing population. This finding is consistent with the results reported by Mahdi et al. [43]. In terms of dimension scores on the scale, nurses scored highest on the “procrastination in physical health promotion” dimension and lowest on the “procrastination in maintaining physical health” dimension. This suggests that nurses procrastinate less when dealing with existing health problems but show a higher tendency to delay proactive health promotion behaviors. This result aligns with the report by Li et al. [44]. It indicates that nurses are more inclined to postpone preventive health behaviors that require active planning and sustained investment, while procrastinating relatively less when addressing manifested health issues. A possible explanation for this phenomenon is that the chronically high workload and fast‐paced nature of clinical nursing work can easily lead to professional fatigue, causing health promotion to be often deprioritized or delayed. Thus, health‐related procrastination may serve as a way to cope with stressors [45]. This suggests that nursing managers should pay attention to the current state of nurses’ health‐related procrastination, assist nurses in developing specific and feasible strategies to initiate health behaviors amidst busy schedules, and thereby promote the reduction of health‐related procrastination.

### 4.2. Correlations between Social Support, Achievement Motivation, and NHRP

The results of this study showed that nurses’ social support was significantly associated with their achievement motivation, which is consistent with the findings of Nasurdin et al. [46], confirming that social support serves as a key resource for enhancing nurses’ work motivation. Social support has been positively correlated with nurses’ job satisfaction and attitude, further supporting its promotive effect on work motivation [47]. Additionally, the results showed a significant negative correlation between nurses’ social support and health‐related procrastination. Adequate social support can enhance coping abilities and self‐efficacy, facilitate positive behavioral changes, improve the regulation of anxiety and stress, promote mental health, and thereby reduce procrastination [48]. Nurses’ achievement motivation was also significantly correlated with health‐related procrastination, which is similar to the research results of Rafii et al. [49]. As nurses with high achievement motivation often exhibit strong self‐regulation, their positive mental state supports active involvement in health management. By doing so, they are able to achieve higher work efficiency and quality [50].

These findings suggest that nursing managers can reduce health‐related procrastination among nurses by strengthening team support and peer encouragement to enhance perceived social support. Furthermore, integrating health goals with personal career development and providing recognition and constructive feedback may help reinforce achievement motivation. This approach can increase nurses’ initiative in managing their health and help decrease health‐related procrastination.

### 4.3. The Mediating Role of Social Support Between Achievement Motivation and NHRP

The results of this study showed that social support played a significant partial mediating role between achievement motivation and NHRP. This suggested that achievement motivation not only directly influenced such behavior but could also affect NHRP indirectly through the mediating pathway of social support. According to Self‐Determination Theory, social support is a key environmental condition that fulfills individuals’ needs for autonomy, competence, and relatedness [51]. Achievement motivation serves as an important internal source of autonomy and competence, while social support provided by the external environment can further satisfy these psychological needs, encouraging nurses to internalize health tasks as self‐identified goals, thereby enhancing self‐regulation and reducing procrastination [52]. Research by Ahlstedt et al. indicated that when nurses perceive support from colleagues and the organization, their higher achievement motivation is more readily translated into identification with tasks, which helps to stimulate positivity, thus reducing procrastination [53].

Furthermore, from the perspective of Conservation of Resources Theory, social support, as a crucial external resource, helps nurses with high achievement motivation conserve psychological resources and reduce depletion [54]. Conversely, insufficient support may lead highly motivated individuals to experience emotional exhaustion, thereby exacerbating procrastination tendencies related to health tasks. Yang et al.’s study on college students demonstrated that social support significantly reduces academic procrastination by enhancing intrinsic motivation and self‐efficacy [55], a mechanism that is also applicable to the clinical nursing context.

These findings suggest that to reduce NHRP, nursing managers should adopt a dual‐track approach. On one hand, efforts should be made to foster a supportive team atmosphere and provide necessary psychological and human resources to strengthen nurses’ psychological capital. On the other hand, fair and timely incentive and recognition mechanisms should be established to continuously activate and sustain nurses’ achievement motivation, effectively translating it into positive health behaviors.

## 5. Conclusions

This study constructed and tested a simple mediation model to explore the relationships among achievement motivation, social support, and NHRP, as well as the mediating role of social support. The results indicated significant correlations among the three variables, with social support playing a partial mediating role between achievement motivation and NHRP. Nurses’ achievement motivation can directly influence their health‐related procrastination and also indirectly affect it through the mediating pathway of social support. From the perspective of positive psychology, this study provides a theoretical basis for strengthening nurses’ health management. It suggests that stimulating nurses’ achievement motivation regarding their own health responsibility and fostering a supportive organizational atmosphere can effectively reduce their health‐related procrastination, thereby laying a foundation for improving overall nursing service quality.

### 5.1. Strengths and Limitations

This study’s primary strengths lie in being the first to focus on health‐related procrastination among nurses, thereby addressing a research gap in this field. Grounded in the triadic reciprocal determinism theory, it constructs an integrated model encompassing achievement motivation, social support, and NHRP, providing a theoretical foundation for interventions related to nurses’ health management. At the same time, the study has certain limitations: the sample was drawn from a single healthcare institution, which may affect the generalizability of the findings; the use of a cross‐sectional design and self‐reported questionnaires limits causal inference and may introduce measurement bias. Future research could adopt multicenter, large‐sample designs, incorporate objective indicators, and employ longitudinal approaches to further validate and extend the findings of this study.

## 6. Implications for Nursing Management

This study reveals the key mediating role of social support between achievement motivation and NHRP, suggesting that nursing managers should implement integrated strategies from a positive psychology perspective. First, through dual pathways of goal motivation and stress counseling, managers should enhance nurses’ positive motivation to strive for success while alleviating anxiety stemming from fear of failure. Second, it is essential to establish a work environment characterized by emotional and informational support, as well as effective peer support systems and communication channels. Lastly, by organically integrating individual motivation guidance with organizational support systems, a synergistic management approach for health promotion can be formed, thereby effectively reducing NHRP and fostering the coordinated development of both their physical and mental well‐being and overall nursing quality.

## Author Contributions

Lan Chen was responsible for guiding the research design and research methods. Haili Zhang and Yi Sheng completed the data collection, analysis, and paper writing and are co‐first authors. Meiqin Hu helped complete the collection of data. Liangyi Cao and Ling Xu were responsible for the content of the pictures and the table.

## Funding

This work was supported by the 2025 Songjiang District Science and Technology Commission Research Project (grant number 2025SJKJGG120) and the 2025‐2026 Hospital Management Innovation Research Project (grant number YNGL‐2025–02).

## Conflicts of Interest

The authors declare no conflicts of interest.

## Supporting Information

Additional supporting information can be found online in the Supporting Information section.

## Supporting information


**Supporting Information 1** Supporting 1. Supporting Information 1, Table1: Dimensions and specific items of the 25‐item nurses’ health‐related procrastination scale (NHRPS) in Chinese and English. Table 1 presents the 25‐item NHRPS in Chinese and English. It covers four dimensions: procrastination in maintaining physical health (items 1–8), procrastination in physical health promotion (items 9–12), procrastination in social and mental health (items 13–21), and procrastination in spiritual health (items 22–25). Responses were rated on a Likert 5‐point scale, ranging from “*never*” (1 point) to “*always*” (5 points).

**Supporting Information 2** Supporting 2. Supporting Information 2, Table1: Normality test results for continuous variables. Table 1 presents the results of the normality tests for the core variables in the manuscript (achievement motivation, social support, and nurses’ health‐related procrastination). The results indicated that the scores for social support and nurses’ health‐related procrastination followed a normal distribution, whereas the scores for the two subscales of achievement motivation did not.

**Supporting Information 3** Supporting 3. Supporting Information 3, Table1: General Characteristics and Univariate Analysis of Nurses’ Health‐Related Procrastination (*n* = 320). Table 2 presents the variables included in the multiple linear regression and their coding. These two tables presented the screening process for the control variables.

## Data Availability

The data that support the findings of the study are available from the corresponding author upon reasonable request.
