# Supplementary material for: The Influence of Achievement Motivation on Nurses’ Health‐Related Procrastination: The Mediating Role of Social Support
Source: J Nurs Manag. 2026 Apr 30;2026:3802852. doi: 10.1155/jonm/3802852 (PMC13131054; doi:10.1155/jonm/3802852)
Supplement: Supplementary file 1 — Supporting Information 1 Supporting 1. Supporting Information 1, Table1: Dimensions and specific items of the 25‐item nurses’ health‐related procrastination scale (NHRPS) in Chinese and English. Table 1 presents the 25‐item NHRPS in Chinese and English. It covers four dimensions: procrastination in maintaining physical health (items 1–8), procrastination in physical health promotion (items 9–12), procrastination in social and mental health (items 13–21), and procrastination in spiritual health (items 22–25). Responses were rated on a Likert 5‐point scale, ranging from “never” (1 point) to “always” (5 points). [file JONM-2026-3802852-s001.docx]

**Table 1. Dimensions and specific items of the 25-item nurses’ health-related procrastination scale (NHRPS) in Chinese and English.**

指导语（Guidance）: 以下条目是指尽管预计结果会更糟，但仍没有任何合理理由时产生的拖延行为。**用于调查您与健康相关的拖延行为现状**。**请您根据实际情况作答，在相应数字上“✓”**（The following items are those that occur without any reasonable reason for the delay and despite expecting to be worse off. To investigate your current health-related procrastination behavior. **Please answer according to the actual situation, in the corresponding number "✓"**.）

| **分级（Rating）：1=从不（never），2=很少（rarely），3=有时（sometimes），4=经常（often）, 5=总是（always）** | | | | | | | |
| --- | --- | --- | --- | --- | --- | --- | --- |
| **N** | **条目** | **Item** | **1** | **2** | **3** | **4** | **5** |
| 1 | 我没有定期免疫接种和检测乙肝抗体滴度，尽管这对护士是必要的 | I delay getting vaccinated and controlling my antibody titers for hepatitis, although these are essential for nurses | 1 | 2 | 3 | 4 | 5 |
| 2 | 在护理操作中，我没有及时运用人体力学原理（如恰当的身体姿势） | I delay adhering to the principles of ergonomics (proper body positions) when providing nursing care | 1 | 2 | 3 | 4 | 5 |
| 3 | 我没有及时进行手卫生，尽管这对护士健康很重要 | I delay my hand hygiene, although it is essential for nurses' health | 1 | 2 | 3 | 4 | 5 |
| 4 | 我没有及时去体检，尽管获取医疗服务很方便 | I delay my medical checkups, although medical services are available | 1 | 2 | 3 | 4 | 5 |
| 5 | 我没有及时就医，尽管就医很方便 | I delay my doctor's visit, although the doctor is available | 1 | 2 | 3 | 4 | 5 |
| 6 | 我没有及时去做已经预约的检查 | I delayed my scheduled test | 1 | 2 | 3 | 4 | 5 |
| 7 | 我没有定期监测健康相关指标（如血压），尽管有可用的设备 | I delay monitoring health-related indicators (such as blood pressure), although devices are available | 1 | 2 | 3 | 4 | 5 |
| 8 | 在护理操作中，我没有及时采取职业防护措施（如戴手套、口罩、护目镜，穿隔离衣） | I delay taking occupational protection measures (gowns, masks, gloves, glasses) when providing nursing care | 1 | 2 | 3 | 4 | 5 |
| 9 | 我没有按时就寝和休息，尽管这对护士健康很重要 | I delay having enough sleep and rest, although they are necessary for nurses' health | 1 | 2 | 3 | 4 | 5 |
| 10 | 我没有按时锻炼身体 | I delay exercising | 1 | 2 | 3 | 4 | 5 |
| 11 | 我推迟执行体重控制计划 | I delay weight control program | 1 | 2 | 3 | 4 | 5 |
| 12 | 我没有及时选择更健康的饮食 | I delay having a healthier diet | 1 | 2 | 3 | 4 | 5 |
| 13 | 出现心理健康问题时，我没有及时寻求帮助和治疗 | I delay seeking help and treatment for my mental health problems | 1 | 2 | 3 | 4 | 5 |
| 14 | 我推迟进行令我开心的活动（如旅行） | I delay activities that make me happy (i.e. travel) | 1 | 2 | 3 | 4 | 5 |
| 15 | 在医院处理使我有压力的病例后，我没有及时释放情绪 | I delay releasing my feelings after dealing with stressful cases in the hospital | 1 | 2 | 3 | 4 | 5 |
| 16 | 我推迟与亲朋好友的见面 | I delay meeting my friends and relatives | 1 | 2 | 3 | 4 | 5 |
| 17 | 我推迟与同事的社交活动 | I delay socializing with my colleagues | 1 | 2 | 3 | 4 | 5 |
| 18 | 我推迟与家人建立亲密关系 | I delay the establishment of intimate relationships with my family | 1 | 2 | 3 | 4 | 5 |
| 19 | 我推迟学习与我的职业相关的新技能和新知识 | I delay learning new skills and acquiring new knowledge related to my profession | 1 | 2 | 3 | 4 | 5 |
| 20 | 我推迟追求能提高我社会地位的事情（如接受更高的教育） | I delay pursuing things that improve my social status (i.e. higher education) | 1 | 2 | 3 | 4 | 5 |
| 21 | 我推迟和同事参与团体活动 | I delay participating in group activities with my colleagues | 1 | 2 | 3 | 4 | 5 |
| 22 | 我没有及时关注自己的精神世界 | I delay attending to my spirituality | 1 | 2 | 3 | 4 | 5 |
| 23 | 我没有及时自我反省、追求生命价值 | I delay my self-reflection and pursuit of life value | 1 | 2 | 3 | 4 | 5 |
| 24 | 我没有及时阅读促进精神健康的书籍 | I delay reading books that promote spiritual health | 1 | 2 | 3 | 4 | 5 |
| 25 | 我没有及时参加能够促进精神健康的活动或仪式 | I delay participating in activities or ceremonies that promote spiritual health | 1 | 2 | 3 | 4 | 5 |
| 总得分 | | Total score |  | | | | |

护士健康相关拖延量表包含25个条目，共四个维度，分别是：与保持身体健康相关的拖延（1~8）、与促进身体健康相关的拖延（9~12）、与社会和心理健康相关的拖延（13~21）和与精神健康相关的拖延（22~25）。每个条目均根据 Likert 5 级评分法评分，从“从不”（1分）到“总是”（5分），总分越高，说明护士的健康相关的拖延行为水平越高。量表总 Cronbach’s α 系数为0.930，信效度良好。

The Nurses' Health-Related Procrastination Scale consists of 25 items distributed across four dimensions: procrastination in maintaining physical health (items 1~8), procrastination in physical health promotion (items 9~12), procrastination in social and mental health (items 13~21), and procrastination in spiritual health (items 22~25). Responses were rated on a Likert 5-point scale, ranging from "never" (1 point) to "always" (5 points). A higher total score indicates a greater degree of health-related procrastination behavior among nurses. The overall Cronbach's α coefficient for the scale was 0.930, demonstrating excellent reliability and validity.
