# Supplementary material for: The Influence of Achievement Motivation on Nurses’ Health‐Related Procrastination: The Mediating Role of Social Support
Source: J Nurs Manag. 2026 Apr 30;2026:3802852. doi: 10.1155/jonm/3802852 (PMC13131054; doi:10.1155/jonm/3802852)
Supplement: Supplementary file 2 — Supporting Information 2 Supporting 2. Supporting Information 2, Table1: Normality test results for continuous variables. Table 1 presents the results of the normality tests for the core variables in the manuscript (achievement motivation, social support, and nurses’ health‐related procrastination). The results indicated that the scores for social support and nurses’ health‐related procrastination followed a normal distribution, whereas the scores for the two subscales of achievement motivation did not. [file JONM-2026-3802852-s002.docx]

TABLE 1: Normality test results for continuous variables.

| **Continuous variables** | **Mean** | **SD** | **Kurtosis** | **Skewness** | **K-S Test (**$\boldsymbol{p}$**)** |
| --- | --- | --- | --- | --- | --- |
| Nurses' health-related procrastination | 10.26 | 0.15 | -0.47 | 0.26 | >0.05 |
| Social support | 8.02 | 0.09 | -0.10 | -0.11 | >0.05 |
| Motive for success | 2.57 | 0.04 | -0.61 | -0.12 | <0.05 |
| Motive to avoid failure | 2.70 | 0.04 | -0.38 | -0.27 | >0.05 |
