# Supplementary material for: The Influence of Achievement Motivation on Nurses’ Health‐Related Procrastination: The Mediating Role of Social Support
Source: J Nurs Manag. 2026 Apr 30;2026:3802852. doi: 10.1155/jonm/3802852 (PMC13131054; doi:10.1155/jonm/3802852)
Supplement: Supplementary file 3 — Supporting Information 3 Supporting 3. Supporting Information 3, Table1: General Characteristics and Univariate Analysis of Nurses’ Health‐Related Procrastination (n = 320). Table 2 presents the variables included in the multiple linear regression and their coding. These two tables presented the screening process for the control variables. [file JONM-2026-3802852-s003.docx]

TABLE 1: General Characteristics and Univariate Analysis of Nurses’ Health-Related Procrastination ($n$ = 320).

| **Nurse characteristics** | **Category** | ***N* (%)** | **Nurses' health-related procrastination** | |
| --- | --- | --- | --- | --- |
|  |  |  | ***t/F*** | ***p*** |
| Gender | Male | 7 (2.2) | 0.415 | > 0.05 |
|  | Female | 313 (97.8) |  |  |
| Age | ≤25 | 49 (15.3) | 12.606 | < 0.001 |
|  | 26-30 | 68 (21.3) |  |  |
|  | 31-40 | 144 (45.0) |  |  |
|  | ≥41 | 59 (18.4) |  |  |
| Type of clinical unit | Internal Medicine | 68 (21.3) | 0.204 | > 0.05 |
|  | Surgery Department | 67 (20.9) |  |  |
|  | Obstetrics and Gynecology | 40 (12.5) |  |  |
|  | Pediatrics Department | 23 (7.2) |  |  |
|  | Emergency and ICU | 81 (25.3) |  |  |
|  | Other | 41 (12.8) |  |  |
| Education level | Diploma in Nursing | 49 (15.3) | 6.780 | < 0.01 |
|  | Bachelor Degree | 260 (81.3) |  |  |
|  | Master Degrees or above | 11 (3.4) |  |  |
| Marital status | Married - | 227 (70.9) | -3.354 | < 0.001 |
|  | Unmarried | 93 (29.1) |  |  |
| Parental status | Has children | 206 (64.4) | -3.735 | < 0.001 |
|  | No children | 114 (35.6) |  |  |
| Professional title | Nurse | 47 (14.7) | 6.458 | < 0.001 |
|  | Nurse practitioner | 173 (54.1) |  |  |
|  | Nurse in charge | 97 (30.3) |  |  |
|  | Associate professor of nursing or above | 3 (0.9) |  |  |
| Years of working | ≤5 | 81 (25.3) | 7.880 | < 0.001 |
|  | 6-10 | 68 (21.3) |  |  |
|  | 11-15 | 58 (18.1) |  |  |
|  | 16-20 | 72 (22.5) |  |  |
|  | ≥21 | 41 (12.8) |  |  |
| Average monthly income | ≤5000 | 15 (4.7) | 3.792 | < 0.01 |
|  | 5001-7000 | 31 (9.7) |  |  |
|  | 7001-9000 | 116 (36.3) |  |  |
|  | 9001-10000 | 105 (32.8) |  |  |
|  | ≥10001 | 53 (16.6) |  |  |
| Certified specialist nurse | Yes | 114 (35.6) | -1.639 | > 0.05 |
|  | No | 206 (64.4) |  |  |

TABLE 2: Variables included in the multiple linear regression and their coding.

| **Independent Variable** | **Assignment** |
| --- | --- |
| X1：Age | ≤25 = (0,0,0,0); 26-30 = (0,1,0,0); 31-40 = (0,0,1,0); ≥41 = (0,0,0,1) |
| X2：Education level | Diploma in Nursing = (0,0,0); Bachelor Degree = (0,1,0); Master Degrees or above = (1,0,0) |
| X3：Marital status | Married = 1，Unmarried = 2 |
| X4：Parental status | Has children = 1; No children = 2 |
| X5：Professional title | Nurse = (0,0,0,0); Nurse practitioner = (0,1,0,0); Nurse in charge = (0,0,1,0); Associate professor of nursing or above = (0,0,0,1) |
| X6：Years of working | ≤5 = (0,0,0,0,0); 6-10 = (0,1,0,0,0); 11-15 = (0,0,1,0,0); 16-20 = (0,0,0,1,0); ≥20 = (0,0,0,0,1) |
| X7：Average monthly income | ≤5000 = (0,0,0,0,0); 5001-7000 = (0,1,0,0,0);  7001-9000 = (0,0,1,0,0); 9001-10000 = (0,0,0,01); ≥10001 = (0,0,0,0,1) |
